# The Efficacies of 1-Methylcyclopropene and Chitosan Nanoparticles in Preserving the Postharvest Quality of Damask Rose and Their Underlying Biochemical and Physiological Mechanisms

**DOI:** 10.3390/biology11020242

**Published:** 2022-02-04

**Authors:** Esmat F. Ali, Ahmed A. Issa, Hatim M. Al-Yasi, Kamel Hessini, Fahmy A. S. Hassan

**Affiliations:** 1 Biology Department Faculty of Science, Taif University, Taif 21944, Saudi Arabia; a.esmat@tu.edu.sa (E.F.A.); a.hissa@tu.edu.sa (A.A.I.); h.alyasi@tu.edu.sa (H.M.A.-Y.); k.youssef@tu.edu.sa (K.H.); 2 Horticulture Department, Faculty of Agriculture, Tanta University, Tanta 31527, Egypt

**Keywords:** antioxidant, chitosan nanoparticles, damask rose, lipid peroxidation, shelf life

## Abstract

**Simple Summary:**

Damask rose is one of the most important aromatic plants that is being used in the perfume, cosmetic and pharmaceutical industries. However, the short blooming period leads to a reduced oil quantity and quality; therefore, preserving the flower shelf life is a crucial step in maintaining the economic viability of this crop. This research aimed to study the effect of 400 mg m^−3^ of 1-methylcyclopropene (1-MCP) and either the pre- or postharvest application of 1% chitosan nanoparticles (CSNPs) on maintaining the quality of damask rose flowers during storage at 4 or 20 °C. The results showed that both treatments were shown to help preserve the quality and extend the shelf life of damask rose. CSNPs were more effective than 1-MCP. Collectively, 1-MCP or CSNPs as eco-friendly applications are recommended as novel and promising approaches for the commercial industry for retaining the quality of damask rose during storage.

**Abstract:**

Preserving the flower shelf life of damask rose is a crucial matter in promoting its economic viability. Chitosan nanoparticles (CSNPs) and 1-methylcyclopropene (1-MCP) may potentially decrease the postharvest loss of several horticultural commodities, but no findings on damask rose have been published. Therefore, the aim of this research was to study the effect of 1-MCP (400 mg m^−3^) and either the pre- or postharvest application of CSNPs (1%) on maintaining the quality of damask rose flowers during storage at 4 or 20 °C. The shelf life of damask rose has been significantly extended, along with a reduction in weight loss due to 1-MCP, CSNPs and pre-CSNP treatments. 1-MCP or CSNP applications have resulted in a higher relative water content, volatile oil, total anthocyanins, total carotenoids, total phenolics and antioxidant activity. Ethylene evolution, H_2_O_2_ generation and malondialdehyde content were significantly decreased due to 1-MCP or CSNPs treatment, and hence, the cell membrane functions have been maintained. The 1-MCP or CSNP-treated flowers have shown higher activities of catalase and ascorbate peroxidase and lower activities of polyphenol oxidase and lipoxygenase in comparison to untreated flowers. Our results showed that the postharvest application of 1-MCP or CSNPs is a very promising method to maintain the postharvest quality of damask rose during storage.

## 1. Introduction

Damask rose (*Rosa damascena* Mill.) is the most important aromatic plant belonging to the *Rosaceae* family. This family includes more than 200 species of the *Rosa* genus that are widely distributed in Europe, Asia, North America and the Middle East [1]. From a commercial point of view, the plant is considered a very important species that is used in the perfume, cosmetic and pharmaceutical industries [2]. The extracted volatile oil of damask rose has been reported to have antiviral, antidiabetic, anti-inflammatory, antioxidant and antibacterial activities [3]. The flower yield, chemical compositions and final quality of the extracted oil are mainly affected by the environmental conditions, cultural and production practices and storage temperatures [4]. Nevertheless, the main factor that adversely affects the quantity and the quality of damask rose volatile oil is the short blooming period, associated with a high flower yield. Therefore, large amounts of flowers call for long extraction cycles, which eventually lead to a reduced final oil quantity and quality [5,6]. Moreover, flowers are generally exposed to oxidative stress caused by mechanical damages during harvest and handling [7]. It is well-known that oxidative stress is mediated by the generation of reactive oxygen species (ROS), which are involved in the cell deterioration process [8]. Storing flowers under unfavorable conditions until extraction also induces lipid peroxidation and the accumulation of malondialdehyde (MDA), a lipid peroxidation marker [9], along with high electrolyte leakage [10,11]. Therefore, preserving the flower quality after harvest until extraction is a crucial step in the production of the volatile oil of damask rose. Some strategies have been used with little success to control the postharvest quality of damask rose, including a cold storage and drying procedure [5]. Unfortunately, cold storage as a sole application was not a beneficial method to protect flowers from chilling injuries that could happen when freshly harvested flowers were stored in a refrigerator [12]. Therefore, it is imperative to find alternative approaches to preserve the quality of damask rose flowers until oil extraction.

The gaseous and nontoxic nature of 1-methylcyclopropene (1-MCP) makes it one of the most potent ethylene perceptions, which blocks the ethylene action by irreversibly binding to the ethylene receptors [13]. As an environmentally friendly ethylene inhibitor, 1-MCP is widely used to delay senescence and preserve the postharvest quality of fruits [14,15,16], leafy vegetables [17,18], herbs [19,20] and cut flowers [21,22,23]. In this context, 1-MCP delayed petal wilting and retarded abscission. Its mode of action is basically related to protecting the flowers from the adverse effects of ethylene via the detoxification of the ROS and, hence, inhibiting lipid peroxidation and preserving the membrane functions [11]. It was reported that 1-MCP reduced ethylene production [15], prevented phenol oxidation [24], increased antioxidant capacity [18] and retained anthocyanins [25]. The combined application of 1-MCP and low temperature considerably reduced H_2_O_2_ and O_2_- generation, electrolyte leakage and the MDA content but increased the total phenol content and antioxidant enzyme activities compared to the control [16,25]. To date, no information is actually yet available either on how the ethylene-related senescence of damask rose is regulated by the use of 1-MCP. Therefore, there is a need for research on preserving the postharvest quality of this economic species during storage until oil extraction.

Chitosan (CS) may also be a good practice candidate for maintaining the quality of damask rose flowers. It is a natural cationic polysaccharide (poly β-(1,4)-N-acetyl-D-glucosamine) and is an interesting biostimulant that has been shown to increase the productivity of several horticultural species [26]. As a natural material extracted from the chitin shells of shrimp and other crustaceans, CS has nontoxic, nonallergenic biodegradable and biocompatible properties [27]. It has also been proven effective in enhancing the biosynthesis of secondary metabolites in several medicinal plants. In this context, CS coating has positively maintained the volatile components and preserved the shelf-life quality of *Tuber melansoporum* [28]. As a bioactive material, CS improved the total phenolics and sugar contents, as well as the antioxidant capacity and, hence, reduced the deleterious effects of oxidative stress [29] that normally occur after the flowers are cut or harvested. Chitosan is commonly used in the preparation of edible coating materials because of its ability to form a semipermeable film, which retards the quality deterioration [30]. Chitosan coating also restricts the exchange of O_2_, CO_2_ and H_2_O_2_ and, hence, improves the permeability of stored products [31]. It has also been used to improve the quality of some cut flowers, such as *Heliconia bihai* [32] and rose [33].

It is unfortunate that conventional CS, as an edible coating, has poor mechanical and permeability properties [34]; therefore, the form of chitosan nanoparticles (CSNPs) was reported to be more effective [35] due to the wide surface area that increases their ability to enter the plant cell, catalyze the plant metabolism and induce high reactivity [36]. In addition, CSNPs are biodegradable stimulants that improve plant growth and the biosynthesis of secondary metabolites and are therefore considered as a new candidate to preserve the postharvest quality of fresh products [12]. Compared to CS, CSNPs are more effective at decreasing the herb surface permeability to water and gases and, therefore, have been successfully used to keep the quality of several horticultural species [37,38]. A positive impact of CSNPs was also observed in some fresh herbs, such as sweet marjoram [39] and basil [12]. Despite using CSNPs to preserve the productivity and quality of several aromatic herbs and fruits, no findings were reported on damask rose flowers, and therefore the present paper is considered to be the first in this area of study.

Although CSNPs can be applied as either preharvest or postharvest applications, studies on the preharvest treatment of CSNPs and their effects to control postharvest quality are still limited. Nonetheless, the preharvest treatment of CS is highly feasible and can be applied around harvest time [40]. Nia et al. [41] reported that CS as a preharvest treatment significantly reduced the decay index, weight loss and malondialdehyde (MDA) content; however, it maintained the overall quality index, firmness, anthocyanin and phenol contents and antioxidant capacity in *Vitis vinifera* L. Accordingly, the preharvest application of CSNPs may also provide considerable protection to damask rose flowers. Very few reports have pointed out the impact of the combined application of 1-MCP and CSNPs on preserving the postharvest quality of damask rose. The main aim of this research was to evaluate how 1-MCP and either a pre- or postharvest application of CSNPs affects the postharvest quality of damask rose flowers, and the underlying physiological and biochemical processes that are involved in regulating the senescence of damask rose flowers in relation to 1-MCP and CSNPs were also studied.

## 2. Materials and Methods

### 2.1. Experiment

A field trial was conducted at a commercial farm (21°26′02.4″ N 40°29′36.9″ E), Taif Governorate, Saudi Arabia during the 2021 season to study the impact of a foliar spray of CSNPs on the postharvest quality of damask rose (*Rosa damascena* Mill. var. *trigentipetala*). The experimental site is located in a highland region (1700 m above sea level). Six-year-old uniform shrubs grown at 2 × 2 m spacings were selected for this study. Entire plants were sprayed in a foliar manner with 1% CSNPs (*m*/*v*) one day before flower harvest, and the control shrubs were sprayed with tap water. On March 15, the flowers were collected in the early morning and were quickly transported at 22 ± 1 °C and 80–85% RH to the laboratory of the Biology Department, Faculty of Science, Taif University. Flowers were then kept in trays and divided into four groups. The first group was CSNP-treated flowers. The second and the third groups were the control flowers that were subjected to postharvest treatments of 1-MCP and CSNPs, respectively. The fourth group was just the control flowers. Half of the control, 1-MCP, CSNP and preharvest CSNP-treated flowers were put in separate trays (≈25 g) and stored at 4 °C and 85–90% RH, while the other half of the flowers was kept at the lab temperature (20 ± 1 °C and 80–85% RH) for 3 days to assess the flower shelf life and quality. This experiment was laid out in a randomized complete block design (RCBD) of 8 treatments with four replicates each, and each replicate contained 4 trays.

### 2.2. 1-MCP Treatment

The powder formulation of SmartFresh^TM^ (0.14% (AgroFresh Solutions, Inc., Philadelphia, PA, USA)) was mixed with distilled water to release 1-MCP [21]. The trays of flowers were kept in a sealed container, and 1-MCP (400 mg m^−3^) was applied for 6 h at 18 ± 1 °C.

### 2.3. CSNPs Preparation and Treatment

The procedure of Fan et al. [42] was used to prepare the CSNPs, based on the ionic gelation of CS (80% deacetylation, 218 kDa Mw (Sigma Aldrich, Burlington, MA, USA)) mixed with tripolyphosphate (TPP) anions. Briefly, a sample of CS (1 g) was dissolved in 100-mL acetic acid (1%) to prepare a 10-mg mL^−1^ solution, and then, the solution was stirred at 60 °C for 30 min and continued overnight at the lab temperature (22 ± 1 °C) for CS dissolution. The solution pH was adjusted to 4.8 using NaOH (1.0 M). To obtain a uniform emulsion, 400 mL of Tween-80 were added to the solution and centrifuged at 10,000× *g* for 10 min. An aqueous solution of Na-TPP (0.75 mg mL^−1^) was gradually added and magnetically stirred with the CS solution at room temperature. The solution was re-centrifuged at 10,000× *g* for 35 min to collect the CSNPs pellets, and the supernatant was preserved at 4 °C until application. The CSNP solution (1%) was then used to foliar spray the plants before harvest. At the postharvest stage, the flowers were dipped in CSNP solution for 10 min.

### 2.4. Shelf Life and Weight Loss Evaluation

Flower freshness and browning were daily evaluated using a visual scale of 0–100% [43] and the shelf-life end when the flowers exhibited more than 50% welting. After 72 h of storage, the weight loss was evaluated. A flower sample (25 g) was initially weighed, and the weight was repeated after 72 h to calculate the weight loss. For the subsequent physiological and biochemical analyses, the samples were kept in −80 °C.

### 2.5. Relative Water Content (RWC)

This was calculated using the following equation of Weatherley [44]:

(W_fresh_ − W_dry_)/(W_turgid_ − W_dry_) × 100, (1)

where W_fresh_, W_turgid_ and W_dry_ are the fresh weight, turgid weight (after saturation in distilled water at 4 °C for 24 h) and dry weight (oven-dried at 70 °C for 48 h), respectively.

### 2.6. Volatile Oil Content and GC-MS Analysis

The hydro-distillation method by a Clevenger-type apparatus [45] was used to extract the volatile oil. The oil percentage was expressed as a fresh weight of flower sample using the following equation:

Volatile oil (%) = (oil volume in the graduated tube/sample fresh weight) × 100.(2)

The extracted oil was dehydrated by Na_2_SO_4_ and stored at 4 °C until gas chromatography-mass spectrometry (GC-MS) analysis. The identification of the volatile components was conducted by Varian GC (CP-3800) and MS (Saturn 2200) equipped with a capillary column (VF-5 ms 30 × 0.25 mm ID and film thickness 0.25 µm). The electron system ionization energy was 70 eV for GC-MS detection. The retention indices (RI) of the peaks were compared with the NIST library of the GC-MS system and the standards to identify the volatile components.

### 2.7. Determination of Total Anthocyanin Content (TAC)

TAC was determined according to Wrolstad et al. [46], with some modifications [47]. Dried samples (0.3 g) were put into a 100-mL beaker that contained 15 mL of the acidified methanol and kept at a lab temperature for 4 h. Filter paper (Whatman No. 1) was used to filter the solution, and the filtrate was then monitored at 530 nm using a spectrophotometer (Pharmacia, LKB-Novaspec II, Uppsala, Sweden). The TAC was calculated by the following equation:

TAC (mg 100 g^−1^ DW) = A × MW × DF × 100/(Ɛ × W), (3)

where A is the absorbance, MW is molecular weight of cyanidin-3-glucoside (449.2 g mol^−1^), DF is the dilution factor, Ɛ is the molar absorptivity (26,900, molar extinction coefficient, in L mol^−1^ cm^−1^, for cyanidin-3-glucoside) and W is the sample weight (g).

### 2.8. Determination of Total Carotenoid Content (TCC)

The TCC was determined according to Dóka et al. [48]. Briefly, a sample of 5 mg from dried fine powder was extracted using 1 mL of acetonitrile (ACN):methanol MeOH):tetrahydrofuran (THF) 50:45:5 by volume in an Eppendorf tube and shaking for 2 h at 150 rpm. The sample was then centrifuged cold at 8163.1× *g* for 5 min at −5 °C. The supernatant was filtered for spectrophotometric measurements. The TCC was estimated from the absorbance monitored at 450 nm on a spectrophotometer (Pharmacia, LKB-Novaspec II). The sample and the standard, diluted in the same solvent mixture, were measured, and the TCC was expressed on a β-carotene basis.

### 2.9. Determination of Total Phenolics Content (TPC)

Dried samples (0.5 g) were ground with a mortar and pestle and put into a glass tube. Then, 10 mL of aqueous methanol was added, and the tube was put into a water bath (80 °C) for 30 min and then cooled. After that, the mixture was centrifuged at 2150× *g* for 30 min at 20 °C, and the supernatant was collected, and its volume was adjusted to 10 mL with aqueous methanol. The TPC was investigated using the Folin–Ciocalteu reagent [49]. Quantification was performed using gallic acid as a standard. The absorbance was measured by a spectrophotometer (Pharmacia, LKB-Novaspec II) at 750 nm. Data were expressed as the mg of gallic acid equivalents (GAE) per g of sample dry weight (mg GAE g^−1^ DW).

### 2.10. Antioxidant Activity (DPPH Assay)

To assess the free radical scavenging activity, the 1,1-diphenyl-2-picrylhydrazil (DPPH) reagent (Sigma-Aldrich, 300267) was used [50]. The extract of the flower sample was soluble in aqueous methanol (85%), and 1.5 mL of DPPH (20 µg mL^−1^) solution was reacted with 0.5 mL of extract and stirred for 5 min in the dark. Then, the decolorizing was monitored at 517 nm against a blank. The DPPH was measured as the inhibition percentage (I%) using the following equation:

I (%) = 100 × (A_blank_ − A_sample_)/A_blank_, (4)

where A_blank_ is the absorbance of control and A_sample_ is the absorbance of the sample at a 30-min reaction. The extract level from generating 50% inhibition was reported as an antiradical activity (IC_50_).

### 2.11. Determination of Ethylene Production

Samples were placed and sealed in 250-mL airtight glass vessels fitted with gas sampling ports. The vessels were maintained at 21 °C and 60–70% RH for 2 h. The gas samples (1 mL) were withdrawn from the vessel’s headspace for ethylene determination. GC-MS (Varian GC CP-3800 and MS Saturn 2200) was used to measure the ethylene production [51], and the results were expressed as (nL g^−1^ h^−1^ FW).

### 2.12. H_2_O_2_ Production

The assaying of H_2_O_2_ production was performed following the method described by Patterson et al. [52]. Briefly, the samples (0.5 g) were homogenized in chilled acetone (6 mL) and centrifuged (12,000× *g*) for 10 min at 4 °C. One microliter of the extract was mixed with 5% Ti(SO_4_)_2_ (0.1 mL) and 0.2 mL of the NH4OH solution, which was centrifuged (3000× *g*) for 10 min. To dissolve the resulting pellet, 4 mL of H_2_SO_4_ (2M) was added. The optical density was then investigated at 412 nm. Various levels of H_2_O_2_ were used to conduct a standard curve for the calibrations, and the results were recorded as µmol g^−1^ FW.

### 2.13. Malondialdehyde Assessment (MDA)

The MDA content was investigated according to Hodges et al. [53] for lipid peroxidation. Each sample (0.2 g) was homogenized in 2-mL trichloroacetic acid (0.1%) and then centrifuged for 15 min at 14,000× *g*. A 2-mL aliquot of the supernatant was added to 5% trichloroacetic acid and 0.5% thiobarbituric acid (3 mL) in hot water (95 °C) and stood for 30 min, following which, the reaction was stopped by cooling the mixture on ice. After that, the mixture was centrifuged for 15 min at 5000× *g*. Finally, the supernatant optical density was monitored at 450, 532 and 600 nm, and the MDA content (μmol mL^−1^) was calculated as follows:

MDA content = 6.45 × (A_532_ − A_600_) − 0.56 × A_450_, (5)

where A_x_ represents the optical density at the subscripted wavelength.

### 2.14. Membrane Stability Index (MSI)

The MSI was assessed according to Sairam et al. [54]. Two samples (0.2 g) were put in two separate flasks (50 mL) contained deionized water (20 mL). The first flask was kept at 40 °C for 30 min, and the second one was put in a water bath (100 °C) for 15 min. the conductivities of both solutions were performed by a conductivity meter and presented as C1 and C2, respectively. The MSI was estimated as ion leakage using the following formula:

MSI (%) = [1 − (C1/C2)] × 100,(6)

### 2.15. Enzyme Activities Determination

The method described by Clairbone [55] was used to determine the activity of CAT (catalase, EC 1.11.1.6). The enzyme extract (0.04 mL) was mixed with 15-mM H_2_O_2_ (0.4 mL) and 50-mM potassium phosphate (pH 7.0) buffer (2.6 mL). The resulting H_2_O_2_ decomposition was monitored by measuring the reduction in absorbance at 240 nm. CAT activity was expressed as U mg^−1^ protein, where one unit was defined by changes of 0.01 H_2_O_2_/60 s.

APX (ascorbate peroxidase, EC 1.11.1.11) activity was assessed according to Nakano and Asada [56]. Each fresh tissue sample (0.1 g) was milled with 0.2 mL of the extraction buffer (3.0-mM EDTA, 0.1-M Na phosphate, pH 7.0, 1.0% Triton X-100 and 1.0% polyvinylpyrrolidone (PVP)) and centrifuged at 10,000× *g* for 20 min. The decline in absorbance at 290 nm, due to enzymatic breakdown, was used to measure the APX activity in the supernatant. The reaction buffer consisted of 0.1-mM H_2_O_2_, 0.5-mM ascorbate, 0.05 mL of extract containing the enzyme and 0.1-mM EDTA mL^−1^; the reaction was performed for 5 min at 25 °C. The coefficient of absorbance at 2.8 mM^−1^ cm^−1^ was used to calculate the activity of APX. One unit of APX enzyme can decompose 1.0 µmol of ascorbate per minute.

Polyphenol oxidase (PPO) activity (EC 1.10.3.2) was studied by the method of Kumar et al. [57]. A powder sample (3 g) was homogenized with 15 mL of sodium phosphate buffer (pH 7.0) at 50 mmol L^−1^ and contained 5% (*w*/*v*) poly-vinylpolypyrrolidone. One unit of PPO activity was defined as the enzyme amount that required causing an increase in the absorbance of 0.001 at 420 nm per minute and was presented as U mg^−1^. To evaluate the lipoxygenase (LOX) activity (EC 1.13.11.12), the methodology of Todd et al. [58] was followed. A sample of 5 g was homogenized in 5 mL of Tris-HCl at 50 mmol L^−1^ (pH 8.0) and contained 10-mmol L^−1^ KCl, 500-mmol L^−1^ sucrose and 0.5-mmol L^−1^ phenylmethylsulfony fluoride. The linoleic acid was used as a substrate, and the enzyme amount required for inducing an increase in the absorption at 234 nm of 0.01 min^−1^ at 25 °C was considered one unit of LOX and expressed as U mg^−1^.

### 2.16. Statistical Analysis

Data were subjected to two-way Analysis of Variance (ANOVA) in the package of SPSS v13.3 (IBM, Armonk, New York, NY, USA). Statistical differences were identified by Duncan’s multiple range test [59], with significance accepted at *p* ≤ 0.05. Values were presented as the means ± SE (*n* = 8).

## 3. Results

### 3.1. Shelf Life and Weight Loss

**Figure 1:**
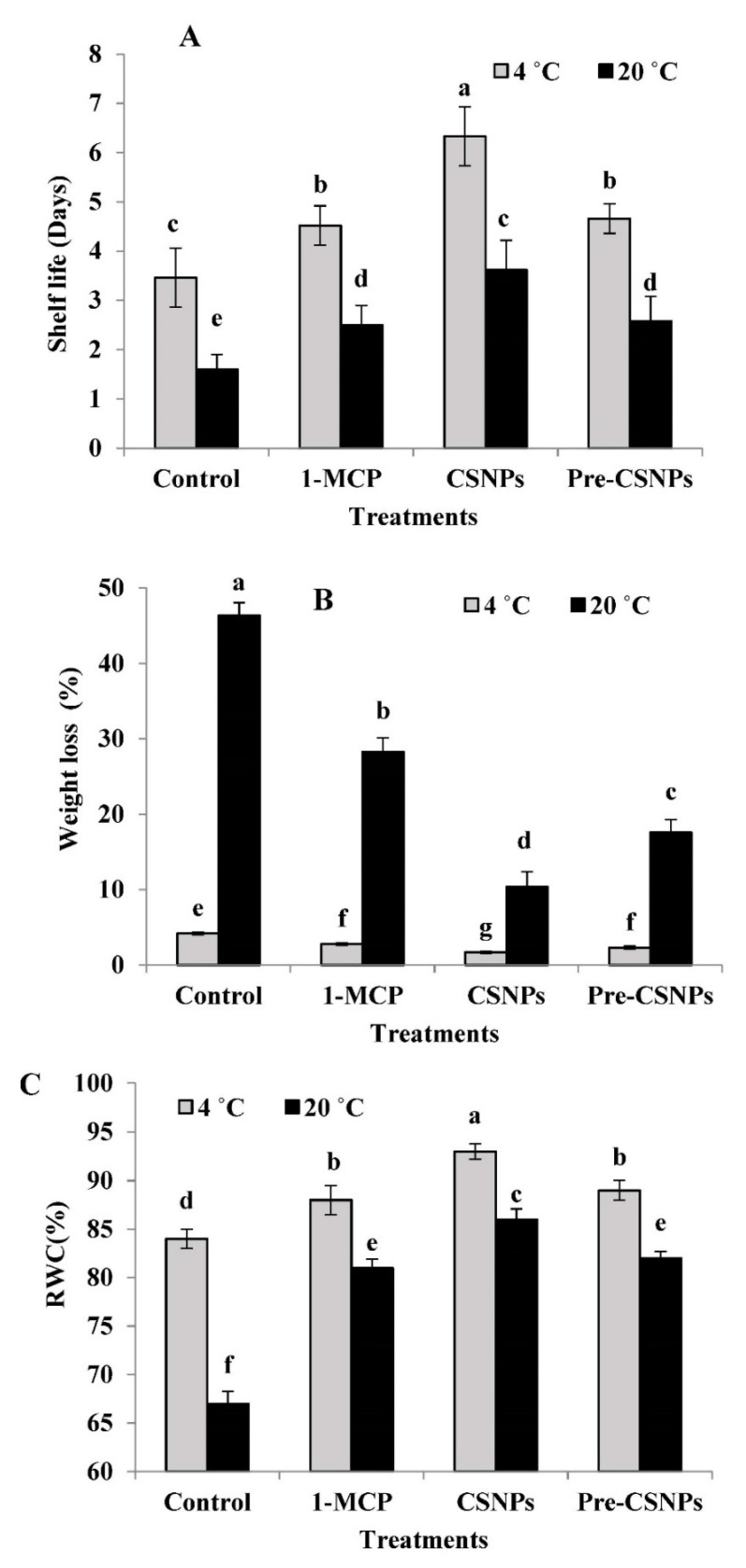
Shelf life (**A**), weight loss (**B**) and relative water content (RWC) (**C**) of damask rose flowers stored at 4 or 20 °C and treated with 1-MCP and pre- and post-chitosan nanoparticles (CSNPs). Values are the means ± SE of two investigations (*n* = 8). Columns with different letters are statistically different at *p* ≤ 0.05.

It is evident from the data in Figure 1A that the shelf life of damask rose was significantly increased due to the 1-MCP, CSNP and pre-CSNP treatments compared to the control. This trend was observed when the flowers were stored at either 4 or 20 °C; however, the shelf life was significantly higher at 4 °C in comparison to 20 °C. The longest shelf life was recorded when the flowers were treated with CSNPs and stored at 4 °C, followed by 1-MCP and pre-CSNPs treatments at the same temperature. Relative to the control, the treatment of CSNPs increased the shelf life by 82.95 and 126.25% at 4 and 20 °C, respectively. Similarly, the 1-MCP, CSNP and pre-CSNP treatments significantly reduced the weight loss of flowers stored at 4 or 20 °C relative to the control (Figure 1B). Flowers stored at 4 °C recorded significantly lower weight loss compared to those stored at 20 °C. The minimum weight loss was observed by applying CSNPs as control flowers, which lost 2.47- and 4.46-fold of their fresh weight compared to those values obtained by the CSNP treatment at 4 and 20 °C, respectively.

### 3.2. Relative Water Content (RWC)

The RWC was significantly higher in flowers treated with 1-MCP, CSNPs and pre-CSNPs in comparison with the control, more so with the storage at 4 °C (Figure 1C). Compared to the control or other treatments, the highest RWC was recorded by CSNPs; however, the difference between 1-MCP and pre-CSNPs was not significant at 4 or 20 °C.

### 3.3. Volatile Oil Content and GC-MS Results

**Figure 2:**
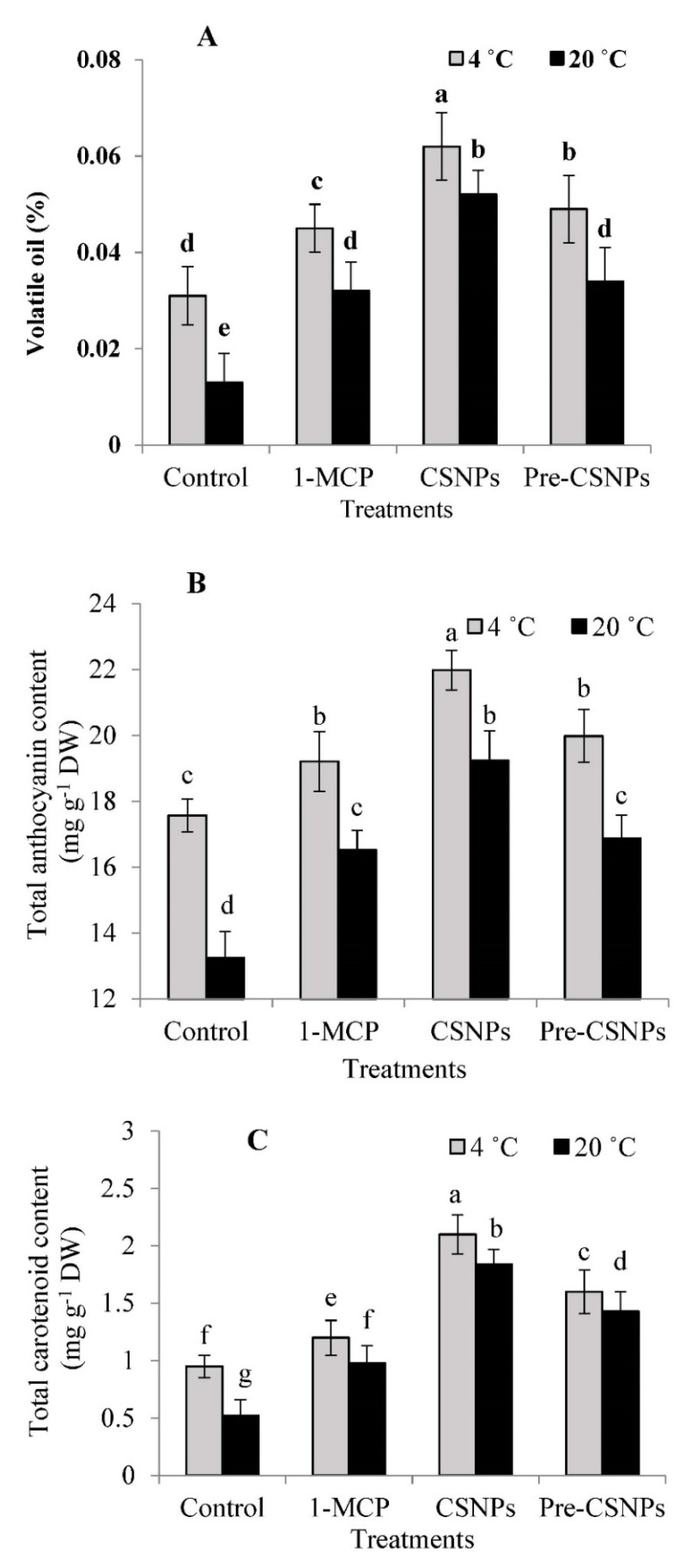
Essential oil percentage (**A**), anthocyanin content (**B**) and carotenoids content (**C**) of damask rose flowers stored at 4 or 20 °C and treated with 1-MCP and pre- and post-chitosan nanoparticles (CSNPs). Values are the means ± SE of two investigations (*n* = 8). Columns with different letters are statistically different at *p* ≤ 0.05.

The treatments of 1-MCP, CSNPs and pre-CSNPs maintained significantly higher volatile oil contents in rose flowers compared to untreated flowers either stored at 4 or 20 °C. Additionally, the volatile oil percentages were significantly higher at 4 °C than those recorded at 20 °C for any treatment or even the control (Figure 2A). The highest oil percentage was recorded by CSNP, followed by pre-CSNP, treatments. After 3 days of storage at 4 and 20 °C, the CSNP treatments resulted in two- and four-fold higher volatile oil percentages compared to the untreated flowers. The GC-MS analysis of the volatile oil indicated that the major components of the volatile oil were linalool, nerol, citronellol, geraniol and nonadecane (Table 1). Those compounds were 55.97% of the identified oil constituents. Generally, a slight improvement was recorded in the volatile oil components in response to applied treatments, more so with the storage at 4 °C. The treatment of the CSNPs almost recorded higher values in this respect than the other treatments.

### 3.4. Total Anthocyanin Content (TAC)

Rose flowers treated with 1-MCP, CSNPs and pre-CSNPs produced significantly higher TAC compared to the control at 4 or 20 °C; however, storage at 4 °C maximized the TAC (Figure 2C). Among the treatments, CSNPs resulted in a significantly higher TAC, even the flowers stored at 4 or 20 °C. In this respect, there were no significant differences between the 1-MCP and pre-CSNPs treatments. Compared to the control, the postharvest treatment of the CSNPs increased the TAC by 25.10 and 45.21% at 4 and 20 °C, respectively.

### 3.5. Total Carotenoid Content (TCC)

The TCC was significantly higher in rose flowers treated with 1-MCP, CSNPs and pre-CSNPs in comparison with the control, more so with the flowers stored at 4 °C. The highest TCC was recorded by the CSNPs, which kept it at considerably higher values than 1-MCP and the pre-CSNPs. However, the difference between 1-MCP and the pre-CSNPs was nonsignificant. The TCC was 2.21- and 3.54-fold higher in the CSNP-treated flowers at 4 and 20 °C compared to the control (Figure 2C).

### 3.6. Total Phenolic Content (TPC)

**Figure 3:**
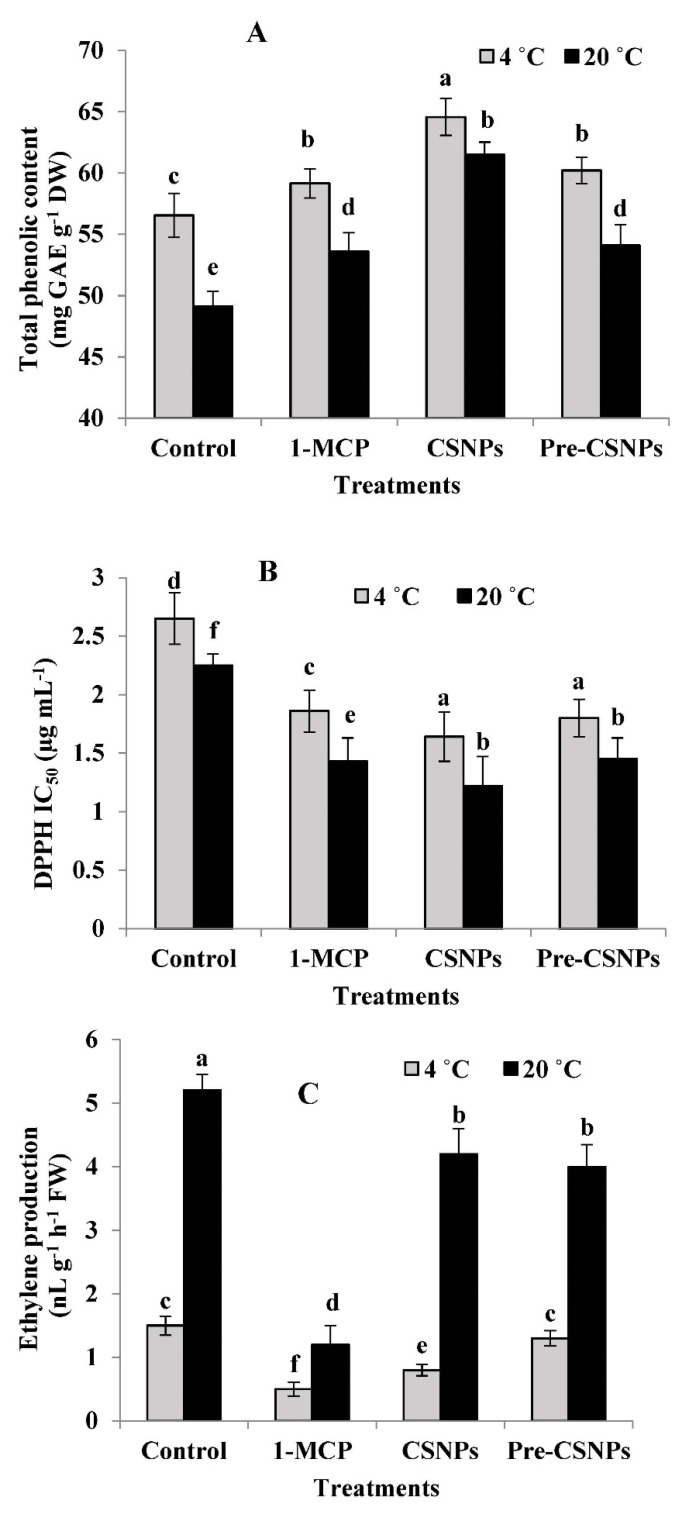
Total phenolics content (**A**), antioxidant activity (**B**) and ethylene production (**C**) of damask rose flowers stored at 4 or 20 °C and treated with 1-MCP and pre- and post-chitosan nanoparticles (CSNPs). Values are the means ± SE of two investigations (*n* = 8). Columns with different letters are statistically different at *p* ≤ 0.05.

The data in Figure 3A show that the TPC was significantly increased due to the 1-MCP, CSNP and pre-CSNP treatments relative to the control. Flower storage at 4 or 20 °C resulted in a similar trend; however, the TPC was significantly higher at 4 °C compared to 20 °C. The highest TPC was recorded in CSNP-treated flowers stored at 4 °C, followed by 1-MCP and pre-CSNP treatments at the same temperature. Relative to the control, the treatment of CSNPs increased the TPC by 14.20 and 25.24% at 4 and 20 °C, respectively, after three days of storage.

### 3.7. Antioxidant Activity

The treatments of 1-MCP, the CSNPs and pre-CSNPs resulted in a significantly higher antioxidant activity; as all treatments considerably lowered the IC_50_ values relative to the control, the flowers were either stored at 4 or 20 °C (Figure 3B). However, storage at 20 °C significantly decreased the antioxidant activity compared to 4 °C. After 3 days of storage, the CSNP-treated flowers had 38.11 and 45.78% higher antioxidant activity than the control at 4 and 20 °C, respectively.

### 3.8. Ethylene Production

The ethylene production was significantly decreased in response to 1-MCP, CSNPs and pre-CSNPs, particularly at 20 °C. The lowest ethylene production was recorded by 1-MCP treatment at 20 °C, with a significant difference compared to the CSNPs or pre-CSNPs. Although, 1-MCP significantly reduced the ethylene production at 4 °C relative to the control, the differences were insignificant compared to the CSNP treatment. Otherwise, there was no significant difference between the pre-CSNPs and control flowers in this respect at 4 °C.

### 3.9. H_2_O_2_ Production

**Figure 4:**
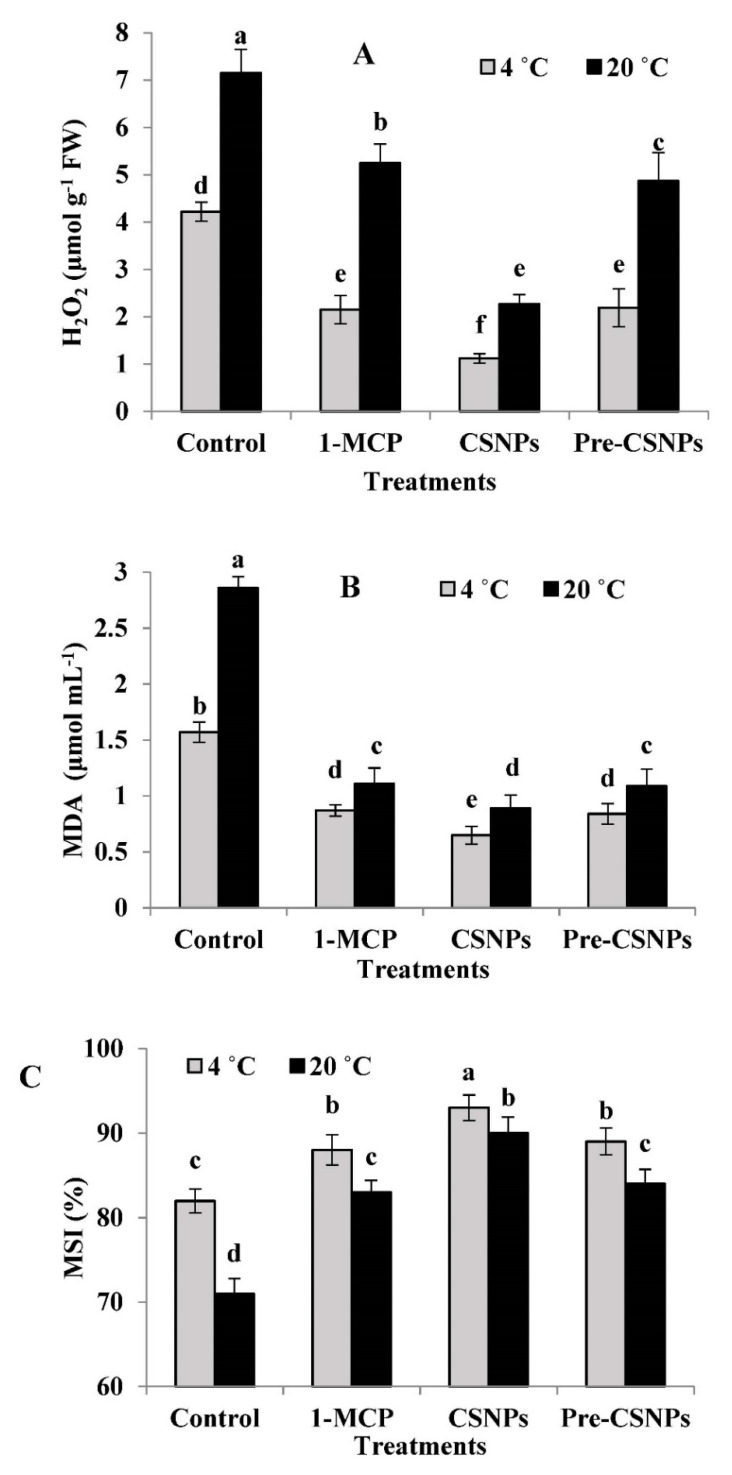
H_2_O_2_ production (**A**), malondialdehyde (**B**) and the membrane stability index (**C**) of damask rose flowers stored at 4 or 20 °C and treated with 1-MCP and pre- and post-chitosan nanoparticles (CSNPs). Values are the means ± SE of two investigations (*n* = 8). Columns with different letters are statistically different at *p* ≤ 0.05.

The flowers treated with 1-MCP, CSNPs and pre-CSNPs produced significantly lower H_2_O_2_ relative to the untreated control at both 4 and 20 °C, especially with the postharvest treatment of the CSNPs (Figure 4A). Otherwise, 1-MCP considerably decreased H_2_O_2_ production compared with the pre-CSNP treatment at 20 °C, but the difference was insignificant at 4 °C. In treated and nontreated flowers, H_2_O_2_ production was significantly lower at 4 than 20 °C. The control flowers produced 3.77- and 3.15-fold higher H_2_O_2_ compared to the CSNP treatment at 4 and 20 °C, respectively.

### 3.10. Malondialdehyde (MDA) Content

1-MCP, the CSNP- and pre-CSNP-treated flowers significantly decreased the MDA accumulation compared to the control, even the flowers were stored at 4 or 20 °C; the impact was greater with the CSNPs than with 1-MCP or pre-CSNPs (Figure 4B). At any storage temperature, there were no significant differences between the 1-MCP and pre-CSNP treatments. CSNP-treated flowers had 2.42- and 3.21-fold lower MDA compared to the untreated flowers at 4 and 20 °C, respectively.

### 3.11. Membrane Stability Index (MSI)

A significantly higher membrane stability was observed in 1-MCP, CSNP- and pre-CSNP-treated flowers compared to that in the control at both storage temperatures. The CSNP treatment resulted in a significantly higher MSI relative to the 1-MCP or pre-CSNP applications (Figure 4C). Otherwise, there were no significant differences between the 1-MCP or pre-CSNP treatments concerning the MSI at 4 or 20 °C. The application of 1-MCP or pre-CSNPs at 20 °C produced no significant difference in the MSI from that of the untreated flowers at 4 °C.

### 3.12. Enzyme Activities Determination

**Figure 5:**
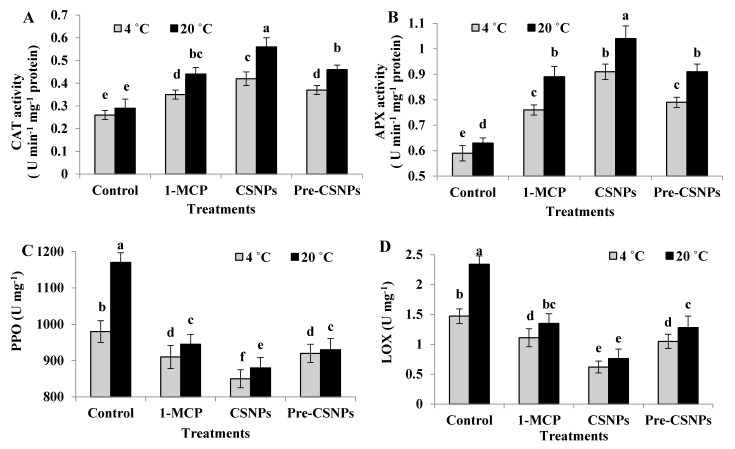
CAT (**A**), APX (**B**), PPO (**C**) and LOX (**D**) of damask rose flowers stored at 4 or 20 °C and treated with 1-MCP and pre- and post-chitosan nanoparticles (CSNPs). Values are the means ± SE of two investigations (*n* = 8). Columns with different letters are statistically different. at *p* ≤ 0.05.

The activities of CAT and APX were significantly increased as a result of the 1-MCP, CSNP and pre-CSNP treatments compared to the control (Figure 5A,B). This trend was observed at both storage temperatures, while the recorded activates at 4 °C were lower than those at 20 °C. Among the treatments, the CSNPs resulted in the highest activities of both enzymes, with significant differences. Otherwise, the activities recorded by 1-MCP were not significant relative to the pre-CSNPs. Flowers treated with CSNPs produced 61.54 and 93.10% higher CAT and 54.24 and 65.08% higher APX activities compared to the control at 4 or 20 °C, respectively. Moreover, 1-MCP, CSNP- and pre-CSNP-treated flowers had significantly lower PPO and LOX enzyme activities compared to the untreated control, more so with CSNPs at 4 °C (Figure 5C,D). Relative to the control, the CSNP treatment significantly lowered the PPO activity by 13.27 and 24.79% when the flowers were stored at 4 or 20 °C. Additionally, the same treatment considerably reduced the LOX activity by 57.82 and 67.52% at both temperatures, respectively.

## 4. Discussion

The current investigation is the first to clarify the effectiveness of 1-MCP and CSNPs to extend the shelf life and preserve the quality of damask rose flowers during storage. The results indicated that the 1-MCP and pre- or postharvest applications of CSNPs significantly (*p* ≤ 0.05) extended the shelf life and decreased the weight loss of rose flowers compared to the control. Additionally, the RWC remained higher in the 1-MCP and CSNP-treated flowers relative to the control. The efficacy of 1-MCP and the CSNPs was more pronounced in flowers stored at 4 °C than those stored at 20 °C. Maintaining the RWC and the flower fresh weight due to the MCP and CSNP applications could be attributed to the capability of CSNPs to isolate the flowers from the outside environment and therefore decreased both the weight loss and the interchange of O_2_ and CO_2_ between the atmosphere and coated flowers [60]. It has been found that weight loss has been ascribed to the reparation process [61] and water loss [19]. The wide surface areas of CSNPs allows better penetration into the cells and high reactivity [36], and hence, it remarkably preserved the fresh weight, RWC and shelf life of damask rose. The effectiveness of CSNPs in keeping the weight and the freshness has been documented in sweet marjoram [39] and basil [12]. Improving the longevity and keeping the fresh weight due to chitosan treatment have been also observed in some cut flowers, such as rose [33] and *Heliconia bihai* [32]. Furthermore, increasing the shelf life and keeping the fresh weight of damask rose flowers in response to 1-MCP treatment may be attributed to its role as an ethylene action inhibitor that irreversibly and competitively acts in blocking the ethylene receptor and, therefore, inhibits the flower senescence [13]. Extending the flower life through maintaining the fresh weight and RWC due to 1-MCP has been reported [21,22].

The importance of damask rose flowers is mainly attributed to the volatile oil content that has biological and medicinal activities, and therefore, retaining the flower volatile oil is a critical factor in preserving the postharvest quality of this economic species [62]. It is worth reporting that the current study is the first work to assess the volatile oil contents in damask rose flowers in response to the 1-MCP and CSNPs treatments. Our results showed that 1-MCP and the CSNPs preserved the volatile oil content during the storage of damask rose flowers. The positive impact of 1-MCP and the CSNPs on the volatile oil content may be due to its effectiveness on keeping the freshness and water contents of tissues, which is required for preserving the secondary metabolites [12]. Consistently, Amer and Shoala [39] found that CSNP treatment improved the volatile oil content and constituents during the storage of sweet marjoram. 1-MCP treatment improved the volatile oil content of coriander leaves during the shelf life, which supports the current findings [19]. Interestingly, the main constituents of volatile oil were preserved during storage, more so at 4 °C due to the 1-MCP and CSNP applications, which adds an impact to their applications in keeping the quality of damask rose flowers. A similar trend has been previously reported in CSNP-treated basil leaves [12] and 1-MCP-treated coriander leaves [19].

It is worth noting here that the TAC, TCC, TPC and antioxidant activity are important postharvest characters in damask rose. In this study, the TAC, TCC, TPC and antioxidant activity (measured by DPPH) were significantly enhanced when the flowers were preharvest-treated with CSNPs or postharvest-treated with 1-MCP and the CSNPs. Phenolic compounds are secondary metabolites that strongly affect the postharvest quality attributes and exhibit antioxidative properties [63]. Secondary metabolites such as flavonoids, anthocyanins and carotenoids play a key role in the pigmentation of flowers [64]. Importantly, CSNPs resulted in higher TPC during the postharvest storage of damask rose relative to the control. Chitosan has been found to stimulate phenolic compound synthesis [29,65,66]. Furthermore, Nia et al. [41] reported that the antioxidant capacity, total phenolics and anthocyanin were considerably improved due to the chitosan coating treatment. Consistent with the current results, chitosan application was able to increase the total phenolics and DPPH radical scavenging activity [29], which were positively associated with the postharvest quality [67]. The positive impact of CSNPs on TAC in damask rose flowers is in accordance with a previous report on the *Heliconia bihai* flower [32].

The treatment of 1-MCP also maintained the TAC, TCC, TPC and antioxidant activity at higher levels in damask rose flowers during storage relative to the untreated flowers. Since the antioxidant activity is closely correlated with the presence of the TPC [68], it has a significant impact on the resistance to oxidative stress-related disorders, and therefore, it should be maintained during the shelf life in healthy products. In agreement with the current results, 1-MCP has been used as an efficient treatment to reduce the total phenolic loss during jujube fruit storage [16]. Maintaining a higher level of antioxidant activity in damask rose due to 1-MCP in the current investigation was similar to the study of Ma et al. [14], who reported that 1-MCP effectively inhibits the reduction of DPPH radical scavenging activity in apples. Similarly, 1-MCP maintained the TAC in carnations [69] and TCC in alstroemeria [70], which support the current results.

In this work, we observed a noticeable effect of 1-MCP and CSNP applications on the reduction of ethylene production of damask rose flowers. Interestingly, this is the first report showing the relation of CSNPs to ethylene production in flowers, particularly on damask rose. The film-forming property and excellent selective permeability of CSNPs to the respiratory gases [12] may participate in decreasing the ethylene production in this study. The production of ethylene is governed by the activity of 1-aminocyclopropane-1-carboxylic acid (ACC) synthase (ACS) and ACC oxidase (ACO). Low O_2_ and high CO_2_ suppress the activity of ACO, which is responsible for converting ACC to ethylene, while high CO_2_ levels prevent ACS autoinduction [71]. Such an effect of high CO_2_ and low O_2_ concentrations on decreasing the ethylene production during storage has been reported in *Pyrus communis* [72]. It has been recently reported that chitosan treatment suppressed ethylene production during the cold storage of kiwifruits, and the reduction of ethylene was accompanied with the suppressed expression of ethylene biosynthesis genes [73]. Similarly, chitosan treatment suppressed the expression of FaACS and FaACO genes that were involved in ethylene biosynthesis, which reduced the softness, increased the shelf life and positively influenced the retention of various quality attributes of strawberries [74].

The 1-MCP treatment also significantly decreased ethylene production compared to the untreated flowers in this study. Decreasing ethylene production due to 1-MCP in the current study may explain the prolonged shelf life of the treated flowers, suggesting that 1-MCP might have a substantial role in controlling the damask rose flower senescence. 1-MCP irreversibly binds with the ethylene receptor on the endoplasmic reticulum membrane to prevent ethylene from combining with its receptor for signal transduction [13]. In this connection, 1-MCP effectively decreased ethylene production and markedly delayed petal senescence in dahlia flowers, suggesting the involvement of ethylene in flower senescence [21]. Additionally, 1-MCP application significantly decreased the activity of ACS and ACO, key enzymes in the ethylene production pathway and, therefore, extending the postharvest life through downregulating the ethylene production in orchids [75] and carnations [22]. Furthermore, inhibiting ethylene synthesis by 1-MCP treatment slows down senescence and retards the senescence-induced destructions of phenolics [16], which supports our results. In agreement with the current report, the effects of 1-MCP in inhibiting the ethylene action and preserving the postharvest quality have been reported in some flowers such as gladiolus [11] and cymbidium [76].

Oxidative stress impaired the functionality of the cell membrane, eventually leading to cell death [11], and it is associated with the diminished quality of postharvest horticultural crops [77]. In the current investigation, the pre/postharvest treatments markedly reduced the H_2_O_2_ production and MDA content and therefore maintained the MSI compared to the control. It is known that ROS play a critical role in cell signal transduction, and excessive ROS damage the cell structure [78]. The CSNP treatment in the current study increased the TPC and antioxidant activity (Figure 3A,B), which resulted in a lower H_2_O_2_ production and MDA content in damask rose flowers. Similarly, a chitosan treatment reduced the lipid peroxidase level and increased the cell membrane integrity of thyme [79]. In the same context, the application of CSNPs was effective in preventing oxidative damage during storage through decreasing MDA and H_2_O_2_ production and preserving the membrane functions [12].

In this study, 1-MCP exhibited a protective effect to the cell membranes, resulting in preserving their integrity, as this treatment reduced the MDA content and H_2_O_2_ generation in rose flowers compared to the untreated ones. In a recent report, a 1-MCP treatment showed higher antioxidant scavenging activity that was able to reduce the ROS levels [17]. Elevated TPC in 1-MCP-treated flowers has been correlated with the impact of 1-MCP on maintaining the membrane integrity, which supports the findings of Fan et al. [80]. It has been found that the browning of tissues is caused by the overproduction of H_2_O_2_ that is declined by the 1-MCP treatment [81], which was similar to the current results. The reduction in MDA and preserving the membrane functions have been elucidated to be reversely proportional with the flower senescence [82,83]. The efficiency of the 1-MCP treatment in retarding the flower senescence via lipid peroxidation reduction has been reported in gladiolus [11] and carnations [22].

Plants have evolved an effective antioxidant mechanism to retard the oxidative stress damage occurred by the overproduction of ROS, which includes enzymatic and nonenzymatic mechanisms to counteract ROS-generated injuries [11,62]. Elevated CAT and APX activities in CSNP-treated flowers compared to the control clearly indicated ROS scavenging in the treated flowers. It is evident that enzymatic antioxidants have a prime role as ROS scavengers, and hence, they are vital to plant defenses against oxidative stresses [43,83]. Preventing cell injury in this study may be due to the functioning of CAT activity to convert H_2_O_2_ to O_2_ and H_2_O [84] and, hence, suppressed ROS accumulation in the flower tissues. These results are in agreement with earlier findings, which revealed that CSNPs could induce the enzymatic antioxidants and H_2_O_2_ scavenging, which improves the membrane integrity and, consequently, enhanced the postharvest quality in loquats [85] and basil [12]. On the other hand, PPO and LOX activities were markedly decreased in CSNP-treated flowers compared to untreated ones, which have considerable value as far as the maintenance of the rose flower quality is concerned during storage. The oxidation of the phenolic compounds by PPO is the main cause of tissue browning, and therefore, PPO induction was observed in the damaged cells [85]. Accordingly, the reduced PPO activity in the current study due to CSNPs most probably participated in flower quality maintenance. The activity of PPO was reduced due to the foliar application of chitosan [40], which was in agreement with our results. The current findings were supported by decreased activity of PPO reported in basil due to CSNP applications [12]. Furthermore, the reduction in LOX activity in CSNP-treated flowers may be ascribed to the impact of CSNPs on reducing H_2_O_2_ production and the MDA content, which resulted in maintaining the membrane integrity relative to the untreated flowers. Petriccione et al. [86] reported that a strong interaction of chitosan (with cationic nature) with the lipids of a membrane likely decreased the affinity of LOX to bind with the membrane fatty acids, leading to LOX activity reduction. The CSNP-induced decline in LOX has been previously reported [12,85].

1-MCP treatment alleviated the oxidative stress induced in rose flowers. That is, it effectively increased the CAT and APX activities and reduced the PPO and LOX activities, which controlled the browning of the rose flowers, which supported the previous report of Xu et al. [25]. Inducing these enzymes is considered an adaptative response to protect cells against oxidative stress [78]. 1-MCP treatment, in the current study, enhanced the antioxidant enzymes, which were able to scavenge the ROS, as indicated by the reduced level of MDA. Amplification of the antioxidant machinery due to 1-MCP treatment has been also reported in gladiolus [11] and carnations [69]. It has been found that 1-MCP treatment reduced the PPO activity in alstroemeria flowers [70]. In agreement with our results, 1-MCP treatment increased the activities of antioxidant enzymes such as CAT and APX, while it was associated with lower PPO and LOX activities [17,87].

## 5. Conclusions

The current investigation is the first report to show that 1-MCP or CSNPs had the capability to preserve the quality and extend the shelf life of damask rose. The CSNPs were more effective than 1-MCP. The positive effects of 1-MCP or the CSNPs were ascribed to decreasing the ethylene production and enhancing the antioxidant defense systems that, in turn, declined H_2_O_2_ production, lipid peroxidation and maintained the membrane functions. Additionally, both treatments maintained higher relative water contents, volatile oil, total anthocyanins and total carotenoids in cut damask rose flowers. In summary, 1-MCP or CSNPs as eco-friendly applications are recommended as novel and promising treatments for the commercial industry for retaining the quality of damask rose during storage.

## Figures and Tables

**Table 1:** GC-MS analysis of damask rose cut flowers stored at 4 or 20 °C and treated with 1-MCP and pre- and post-chitosan nanoparticles (CSNPs).

No.	RI	Compound	Control (4 °C)	Control (20 °C)	1-MCP (4 °C)	1-MCP (20 °C)	CSNPs (4 °C)	CSNPs (20 °C)	Pre-CSNPs (4 °C)	Pre-CSNPs (20 °C)
			Relative (%)							
1.	1022	α-Pinene	3.47	3.45	3.52	3.51	3.56	3.54	3.49	3.46
2.	1136	β-Pinene	0.54	0.55	0.59	0.58	0.61	0.63	0.58	0.56
3.	1174	Myrcene	1.86	1.83	1.92	1.90	1.98	1.96	1.85	1.83
4.	1494	Linalool	6.98	6.92	7.11	7.08	7.22	7.19	7.14	7.12
5.	1511	*cis*-Rose oxide	0.65	0.62	0.69	0.66	0.75	0.73	0.68	0.69
6.	1537	Phenyl ethyl alcohol	2.54	2.49	2.65	2.60	2.69	2.67	2.65	2.63
7.	1541	*trans*-Rose oxide	0.56	0.54	0.61	0.59	0.64	0.63	0.58	0.57
8.	1552	Terpinen-4-ol	1.19	1.17	1.25	1.21	1.24	1.22	1.19	1.18
9.	1574	α-Terpineol	2.47	2.45	2.57	2.54	2.59	2.58	2.56	2.52
10.	1586	Nerol	7.48	7.79	7.53	7.50	7.59	7.52	7.49	7.43
11.	1657	Heptadecane	1.44	1.40	1.46	1.45	1.54	1.51	1.52	1.49
12.	1688	Citronellol	18.86	18.75	18.91	18.88	18.97	18.90	18.89	18.81
13.	1701	Geraniol	15.54	15.51	15.66	15.62	16.11	15.99	15.87	15.80
14.	1714	Geranial	2.83	2.79	2.96	2.85	3.08	2.99	2.97	2.92
15.	1732	Eugenol	1.45	1.46	1.49	1.47	1.53	1.54	1.50	1.47
16.	1739	Geranyl acetate	0.92	0.88	0.94	0.91	0.99	0.98	0.97	0.98
17.	1747	Methyl eugenol	1.29	1.25	1.33	1.31	1.32	1.33	1.36	1.34
18.	1752	α-Guaiene	1.19	1.16	1.18	1.19	1.25	1.26	1.22	1.19
19.	1794	Caryophyllene oxide	0.46	0.48	0.51	0.49	0.46	0.43	0.47	0.45
20.	1806	Octadecane	0.42	0.39	0.44	0.38	0.43	0.44	0.47	0.43
21.	1812	Nonadecene	2.86	2.81	2.89	2.87	2.88	2.89	2.81	2.82
22.	1819	Nonadecane	7.11	6.99	7.18	7.15	7.21	7.19	7.05	6.99
23.	1984	Heneicosane	1.22	1.24	1.28	1.26	1.29	1.24	1.27	1.28

## Data Availability

Not applicable.
